# Draft genome sequences of *Epichloë bromicola* strains isolated from *Elymus ciliaris*

**DOI:** 10.1128/mra.00317-24

**Published:** 2024-09-09

**Authors:** Atsushi Miura, Sayaka Imano, Akira Ashida, Ikuo Sato, Sotaro Chiba, Aiko Tanaka, Maurizio Camagna, Daigo Takemoto

**Affiliations:** 1 Graduate School of Bioagricultural Sciences, Nagoya University, Nagoya, Japan; Rochester Institute of Technology, Rochester, New York, USA

**Keywords:** endophyte, *Epichloë bromicola*, *Elymus ciliaris*

## Abstract

*Epichloë* species are endophytic fungi that systemically colonize grass species. Here, we report the genome sequences of *Epichloë bromicola* strains HS and DP isolated for the first time from *Elymus ciliaris* in Nagoya, Japan.

## ANNOUNCEMENT

**Fig 1:**
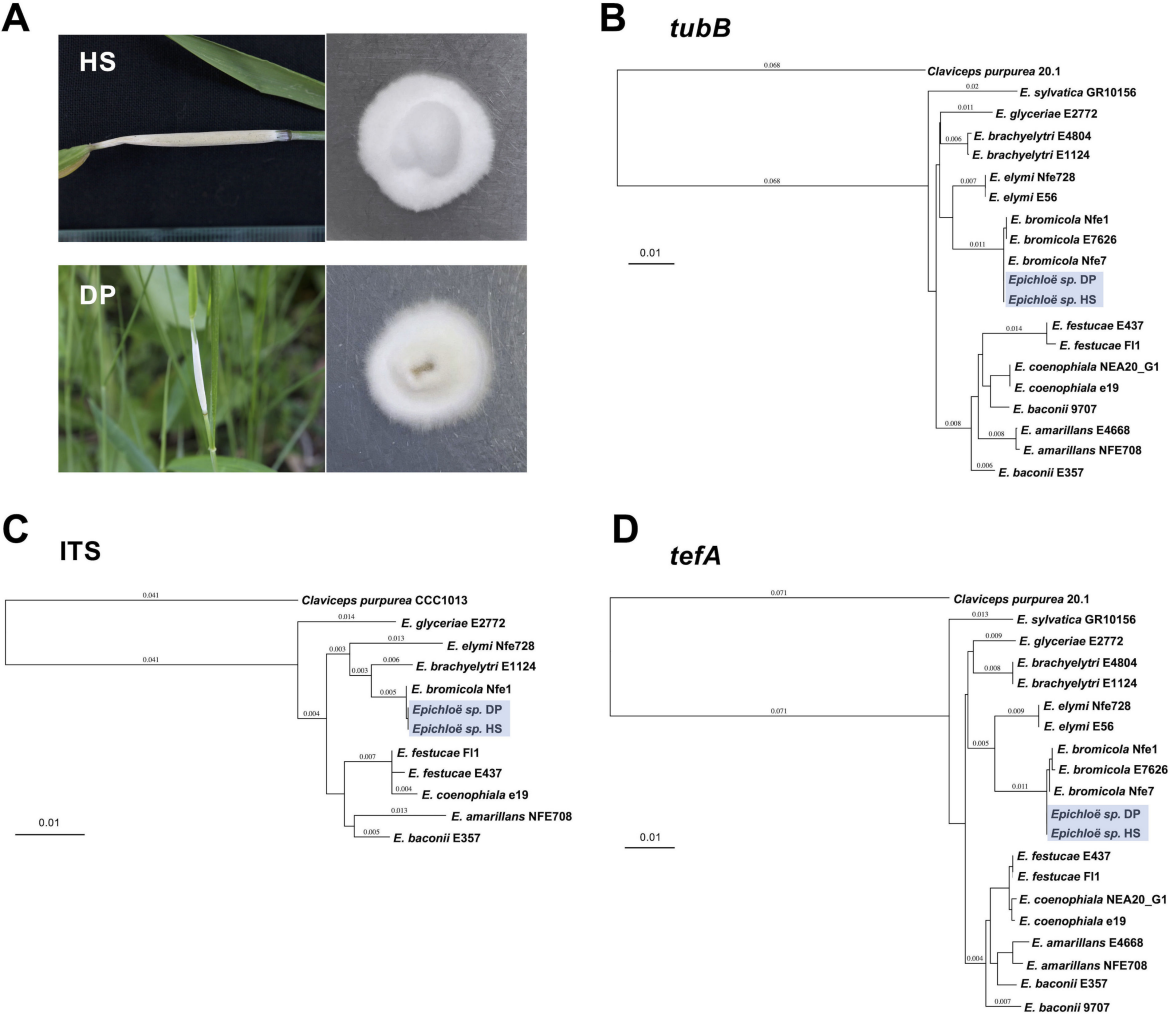
(A) (left) Stroma of *Epichloë bromicola* strains HS and DP formed on pseudostems of *Elymus ciliaris*. (right) Colony of *E. bromicola* strains grown on PDA. (B–D) Phylogenetic analyses of strains HS and DP with *Epichloë* species based on the sequences of *tubB* (B), internal transcribed spacer (ITS) (C), and *tefA* (D). Phylogenetic analysis was performed using neighbor-joining method (15) using MacVector version 18.6.4 (Macvector inc, Apex, NC, USA) with the default setting. Sequences of *Claviceps purpuria* strain 20.1 (*tubB* and *tefA*) or strain CCC1013 (ITS) were used as the outgroup. The scale bar corresponds to 0.1 estimated nucleic acid substitutions per site.

*Epichloë* is a genus belonging to Ascomycota fungi that are obligate symbionts of cool-season grasses. This group of fungi is being studied extensively for its advanced infection mechanisms on host plants and the production of bioactive substances that protect the host plants from insect and animal herbivory, pathogen infection, drought, and other biotic and abiotic stresses (1–6). Here, we report the draft genome sequences of *Epichloë bromicola* strains HS and DP from *Elymus ciliaris* [a wheatgrass native to east Asia (7)] grown near Hoshigaoka Station (HS, geographic coordinates 35.1627, 136.9863) and in Daiman Park (DP, 35.1588, 136.9953) in Nagoya, Japan. Sexual *Epichloë* spp. typically form stroma on pseudostems of the host plants and suppresses the maturation of host inflorescences, which is called “choke disease” (1, 2). *E. bromicola* strains HS and DP were isolated from pseudostems of *E. ciliaris* with stroma formation (Fig. 1A). Pseudostems of *E. ciliaris* were surface sterilized and set on PDA, and emerged hyphae were isolated on PDA using a previously described method (8). When grown on PDA, *E. bromicola* forms white and fluffy colonies (Fig. 1B), that exhibit relatively slow radial growth (approx. 1.6 cm/week for both strains). Both strains were grown on PDA for 7 days, then subcultured in liquid PDB at 23°C on a rotary shaker at 120 rpm for 4 days for genomic DNA extraction using NucleoSpin Plant II (Takara Bio, Japan) according to the manufacturer’s instructions. Whole-genome sequencing libraries were prepared with MGIEasy FS PCR-Free DNA Library Prep Set (MGI, Cat. No. 1000013454) as per the manufacturer’s instructions and sequenced on DNBSEQ-G400RS (MGI) using DNBSEQ-G400 High-throughput Sequencing Set with 2 × 150 bp read length. Unless otherwise noted, the following data processing and analyses were performed using the application’s default settings. After quality filtering and trimming with fastp v0.22.0 with options to remove adaptors and low-quality reads (9), paired-end data sets were assembled *de novo* using SPAdes v3.15.3 using the -careful option (10). Assembly quality was assessed with QUAST v4.4 (11). The summary of genome assemblies is shown in Table 1. Prediction of the protein-encoding genes was performed with BRAKER3 (12) using the annotated protein sequences of ascomycota_odb10 (13) as input for model hints. The qualities of the assembled genomes were estimated using benchmarking universal single-copy orthologs (BUSCO) using the ascomycota_odb10 data set. Using the Ascomycota data set, the protein sets of HS and DP had BUSCO scores of 98.7% and 98.6% and a total of 1,683 and 1,682 complete BUSCOs for both strains, respectively (Table 1). Based on the phylogenetic analysis using *TefA*, *TubB*, and ITS sequences of *Epichloë* species (14), strains DP and HS were identified as *E. bromicola* (Fig. 1B through D).

**TABLE 1:** Summary of *Epichloë bromicola* genome assemblies and annotations

Strain	Host plant	No. of filtered paired reads	N50	GC contents (%)	No. of total bases (bp)	No. of contigs	Sequencing coverage	No. of genes	Results from BUSCO analysis with data set ascomycota_odb10			
									Complete BUSCOs (%)	No. of BUSCOs	No. of missing BUSCOs	No. of fragmented BUSCOs
HS	*Elymus ciliaris*	66,108,990	46,320	48.5	28,005,820	3,120	703	7,682	98.6	1,682	13	11
DP	*Elymus ciliaris*	60,247,876	47,884	49.0	27,262,061	2,503	658	7,580	98.8	1,684	12	10

## Data Availability

The *E. bromicola* genome sequences were deposited at DDBJ/EMBL/GenBank under accession nos. BAAFGZ010000001 to BAAFGZ010002503 (strain DP) and BAAFHA010000001 to BAAFHA010003120 (strain HS). The raw sequencing reads have been submitted to the DDBJ Sequence Read Archive (DRA) under the accession numbers DRR542286 (DP) and DRR542287 (HS).

## References

[1] Scott B. 2001. Epichloë endophytes: fungal symbionts of grasses. Curr Opin Microbiol 4:393–398. doi:10.1016/s1369-5274(00)00224-1 11495800

[2] Schardl CL. 2001. Epichloë festucae and related mutualistic symbionts of grasses. Fungal Genet Biol 33:69–82. doi:10.1006/fgbi.2001.1275 11456460

[3] Purev E, Kondo T, Takemoto D, Niones JT, Ojika M. 2020. Identification of ε-Poly-L-lysine as an antimicrobial product from an Epichloë endophyte and isolation of fungal ε-PL synthetase gene. Molecules 25:1032. doi:10.3390/molecules25051032 32106587 PMC7179176

[4] Kayano Y, Tanaka A, Takemoto D. 2018. Two closely related Rho GTPases, Cdc42 and RacA, of the en-dophytic fungus Epichloë festucae have contrasting roles for ROS production and symbiotic infection synchronized with the host plant. PLoS Pathog 14:e1006840. doi:10.1371/journal.ppat.1006840 29370294 PMC5785021

[5] Saikia S, Takemoto D, Tapper BA, Lane GA, Fraser K, Scott B. 2012. Functional analysis of an indole-diterpene gene cluster for lolitrem B biosynthesis in the grass endosymbiont Epichloë festucae. FEBS Lett 586:2563–2569. doi:10.1016/j.febslet.2012.06.035 22750140

[6] Takemoto D, Kamakura S, Saikia S, Becker Y, Wrenn R, Tanaka A, Sumimoto H, Scott B. 2011. Polarity proteins Bem1 and Cdc24 are components of the filamentous fungal NADPH oxidase complex. Proc Natl Acad Sci U S A 108:2861–2866. doi:10.1073/pnas.1017309108 21282602 PMC3041104

[7] Hu Q, Yan C, Sun G. 2013. Phylogenetic analysis revealed reticulate evolution of allotetraploid Elymus ciliaris. Mol Phylogenet Evol 69:805–813. doi:10.1016/j.ympev.2013.06.023 23831560

[8] Christensen MJ, Bennett RJ, Schmid J. 2002. Growth of Epichloë / Neotyphodium and p-endophytes in leaves of Lolium and Festuca grasses. Mycol Res 106:93–106. doi:10.1017/S095375620100510X

[9] Chen S, Zhou Y, Chen Y, Gu J. 2018. fastp: an ultra-fast all-in-one FASTQ preprocessor. Bioinformatics 34:i884–i890. doi:10.1093/bioinformatics/bty560 30423086 PMC6129281

[10] Bankevich A, Nurk S, Antipov D, Gurevich AA, Dvorkin M, Kulikov AS, Lesin VM, Nikolenko SI, Pham S, Prjibelski AD, Pyshkin AV, Sirotkin AV, Vyahhi N, Tesler G, Alekseyev MA, Pevzner PA. 2012. SPAdes: a new genome assembly algorithm and its applications to single-cell sequencing. J Comput Biol 19:455–477. doi:10.1089/cmb.2012.0021 22506599 PMC3342519

[11] Gurevich A, Saveliev V, Vyahhi N, Tesler G. 2013. QUAST: quality assessment tool for genome assemblies. Bioinformatics 29:1072–1075. doi:10.1093/bioinformatics/btt086 23422339 PMC3624806

[12] Gabriel L, Brůna T, Hoff KJ, Ebel M, Lomsadze A, Borodovsky M, Stanke M. 2024. BRAKER3: fully automated genome annotation using RNA-seq and protein evidence with GeneMark-ETP, AUGUSTUS and TSEBRA. bioRxiv:2023.06.10.544449. doi:10.1101/2023.06.10.544449 PMC11216308 38866550

[13] Manni M, Berkeley MR, Seppey M, Simão FA, Zdobnov EM. 2021. BUSCO update: novel and streamlined workflows along with broader and deeper phylogenetic coverage for scoring of eukaryotic, prokaryotic, and viral genomes. Mol Biol Evol 38:4647–4654. doi:10.1093/molbev/msab199 34320186 PMC8476166

[14] Quenu M, Treindl AD, Lee K, Takemoto D, Thünen T, Ashrafi S, Winter D, Ganley ARD, Leuchtmann A, Young CA, Cox MP. 2022. Telomere-to-telomere genome sequences across a single genus reveal highly variable chromosome rearrangement rates but absolute stasis of chromosome number. J Fungi (Basel) 8:670. doi:10.3390/jof8070670 35887427 PMC9318876

[15] Saitou N, Nei M. 1987. The neighbor-joining method: a new method for reconstructing phylogenetic trees. Mol Biol Evol 4:406–425. doi:10.1093/oxfordjournals.molbev.a040454 3447015

